# Genotyping by PCR and High-Throughput Sequencing of Commercial Probiotic Products Reveals Composition Biases

**DOI:** 10.3389/fmicb.2016.01747

**Published:** 2016-11-03

**Authors:** Wesley Morovic, Ashley A. Hibberd, Bryan Zabel, Rodolphe Barrangou, Buffy Stahl

**Affiliations:** ^1^Genomics and Microbiome Science, DuPont Nutrition & HealthMadison, WI, USA; ^2^Department of Food, Bioprocessing and Nutrition Sciences, North Carolina State UniversityRaleigh, NC, USA

**Keywords:** probiotics, labeling, testing and assessment, Lactobacillus, Bifidobacterium, multiplex PCR, taxonomy, high-throughput nucleotide sequencing

## Abstract

Recent advances in microbiome research have brought renewed focus on beneficial bacteria, many of which are available in food and dietary supplements. Although probiotics have historically been defined as microorganisms that convey health benefits when ingested in sufficient viable amounts, this description now includes the stipulation “well defined strains,” encompassing definitive taxonomy for consumer consideration and regulatory oversight. Here, we evaluated 52 commercial dietary supplements covering a range of labeled species using plate counting and targeted genotyping. Strain identities were assessed using methods recently published by the United States Pharmacopeial Convention. We also determined the relative abundance of individual bacteria by high-throughput sequencing (HTS) of the 16S rRNA sequence using paired-end 2 × 250 bp Illumina MiSeq technology. Using these methods, we tested the hypothesis that products do contain the quantitative and qualitative list of labeled microbial species. We found that 17 samples (33%) were below label claim for CFU prior to their expiration dates. A multiplexed-PCR scheme showed that only 30/52 (58%) of the products contained a correctly labeled classification, with issues encompassing incorrect taxonomy, missing species, and un-labeled species. The HTS revealed that many blended products consisted predominantly of *Lactobacillus acidophilus* and *Bifidobacterium animalis* subsp. *lactis*. These results highlight the need for reliable methods to determine the correct taxonomy and quantify the relative amounts of mixed microbial populations in commercial probiotic products.

## Introduction

Whereas microbiology has historically focused on pathogens and infectious agents, recent efforts have established the importance that microbiomes in general and beneficial microbes in particular play in promoting and maintaining human health (Turnbaugh et al., 2007; Human Microbiome Consortium, 2012). The benefits of health-promoting bacteria have fueled several investigations establishing the genetic and phenotypic basis for probiotic functionalities (Papadimitriou et al., 2015). The International Scientific Association of Probiotics and Prebiotics defines products containing probiotics as those that “deliver live microorganisms with a suitable viable count of well-defined strains with a reasonable expectation of delivering benefits for the well-being of the host” (Hill et al., 2014), expanding the FAO/WHO definition to include strain-level taxonomy.

The global probiotic industry continues to grow and product introductions rely on the conformation to guidance by local regulatory agencies. Specifically required from several different regulatory bodies (Hammett, 2008; Health Canada, 2015) is accurate labeling of consumer products containing live microbials with species-level identity and viability. Probiotic benefits are typically attributed to specific strains, for which safety and efficacy must be established (Branton et al., 2011; Pariza et al., 2015). In some cases, it is necessary to determine and compare the complete genomes of isolates to accurately identify and distinguish particular genotypes using high-resolution nucleic acid analyses, as beneficial metabolic effects are attributed to these key differences in strains (Briczinski et al., 2009; Broadbent et al., 2012; Ruiz-Moyano et al., 2013). Additionally, strains must be present in sufficient viable quantities to confer a probiotic effect, which varies based on consumer and desired effect (Reid et al., 2001; Leyer et al., 2009). The traditional ISO-approved method of determining viable cell count is by serial dilution and selectively culturing cells to result in colony forming units (CFU) per gram or milliliter (International Organization for Standardization, 2003). While product packaging provides the CFU level content, these are most often reported as the total CFU of a dose and not of individual species or strains. Furthermore, CFU are sometimes measured at time of manufacture although they are known to decrease over time depending on environmental stressors and strain characteristics (Sanders et al., 2014). Indeed, maintaining viability over the course of storage is a major challenge and focus for the probiotics industry.

**Figure 1:**
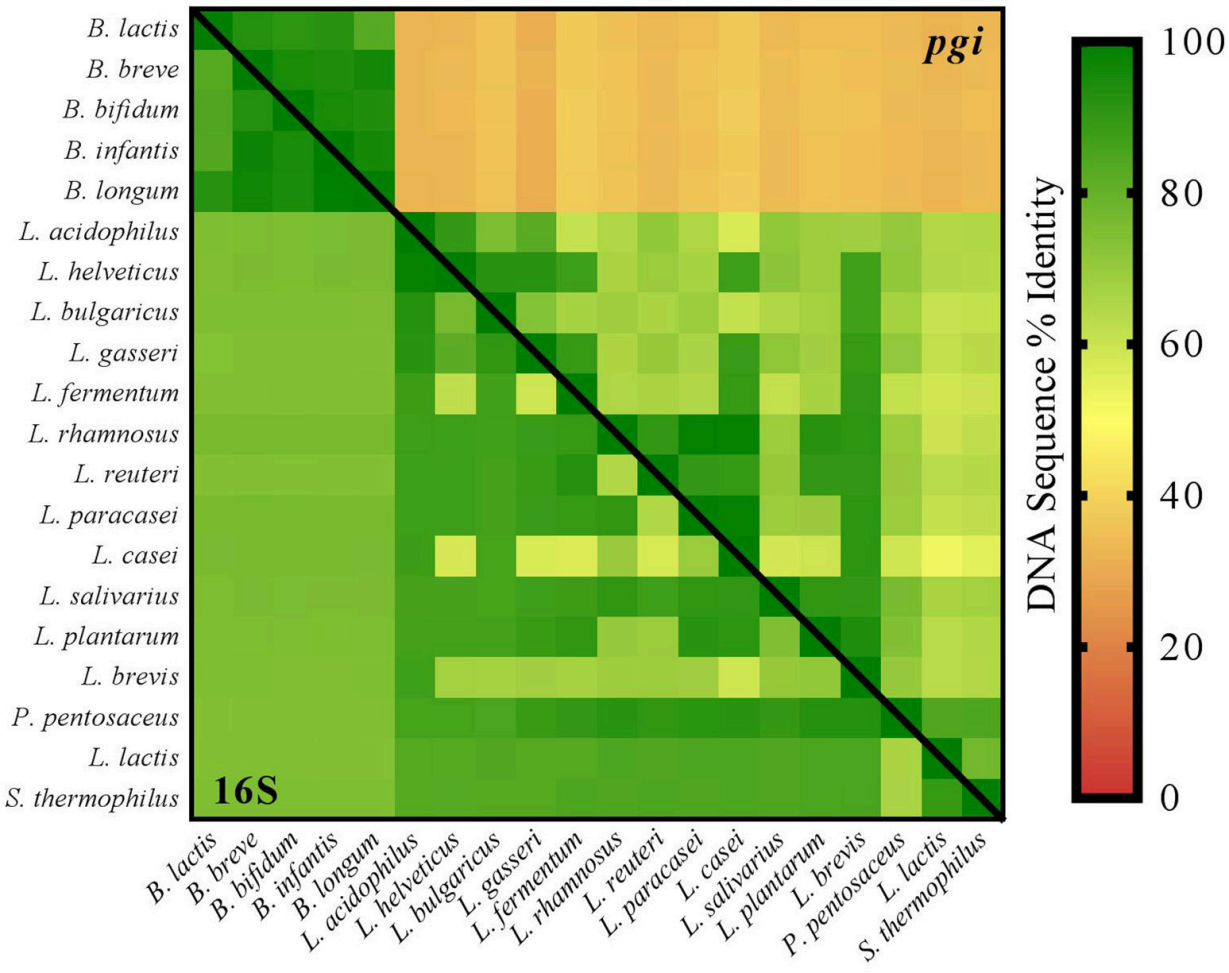
**Sequence homology of the 16S rRNA and the *glucose-6-phosphate isomerase* sequences in commercial probiotics**. Gene sequences for the 16S rRNA and *pgi* genes were aligned separately for all 20 organisms listed using the Geneious alignment algorithm. The resulting percent identity matrices were combined into one table and visualized by heat map using Prism 7.01. The overall sequence similarity is higher in the 16S rRNA genes than the *pgi* genes, which presents opportunities for higher resolution assays.

Advances in sequencing technologies, assembly, and annotation have enabled the scientific community to determine the complete genomes of probiotic strains (Altermann et al., 2005; Stahl and Barrangou, 2013), allowing the development of genotyping methods (Barrangou et al., 2009; Barrangou and Horvath, 2012), and providing unequivocal insights into the proper taxonomy of broadly used commercial strains (Makarova et al., 2006; Briczinski et al., 2009; Loquasto et al., 2013; Milani et al., 2013; Holzapfel and Wood, 2014; Lugli et al., 2014). Trends regarding the formulation of increasingly efficacious and complex blends of multiple probiotics in food and dietary supplements demand the development of high-resolution, yet affordable methods that enable the determination of bacterial counts, and their classification for proper labeling. Some surveys of commercial probiotics have been reported previously, in which authors tested congruence with label claim for phylogenetic identity and CFU counts (Lewis et al., 2015; Patro et al., 2016). Several reports analyzing probiotic claims at the species level focus on ribosomal-based methods. Arguably the gold-standard in prokaryotic taxonomic identification, the 16S rRNA gene contains homologous and polymorphic sequence regions that can be leveraged in techniques including PCR (Angelakis et al., 2011) and subsequent restriction digest banding (Moreira et al., 2005) to affirm taxonomic classification. By combining 16S rRNA gene PCR with High-Throughput Sequencing (HTS) techniques, the relative abundance of bacteria in a sample can be examined in the form of sequence reads (Caporaso et al., 2011). Indeed, this strategy has been revolutionary in assessing microbiomes in many sample types (Cho and Blaser, 2012; De Leoz et al., 2015; Forssten et al., 2015; Butteiger et al., 2016). There are, however, known limitations to using 16S rRNA sequences, including the presence of multiple heterogeneous copies within a single genome and high sequence similarity between species and sub-species (Dahllöf et al., 2000; Mohkam et al., 2016). This is a known challenge for probiotic genera, notably *Bifidobacterium* and *Lactobacillus* (Milani et al., 2014; Sun et al., 2015). In addition to ribosomal genes, whole genome sequences reveal many other conserved genes that offer higher resolution genotyping opportunities (Figure 1). One such gene is *glucose-6-phosphate isomerase* (*pgi*; EC: 5.3.1.9), a single copy gene whose enzyme catalyzes the important reversible reaction of D-glucose-6-phosphate to D-fructose-6-phosphate in the pentose phosphate pathway and glycolysis (Kanehisa et al., 2016). The aforementioned pathways are conserved biochemical cornerstones of most bacteria, and can actually serve as phylogenetic biomarkers (Brandt and Barrangou, 2016). Furthermore, these genes are widespread, well-annotated, and can be leveraged for taxonomic applications.

Typically, the genus and species binomial nomenclature, together with a total or species-attributed CFU count, are reported on the label of probiotic products. Most probiotic dietary supplement products contain a blend of strains representing various combinations of bacterial genera and species, occasionally including a particularly well-documented strain, formulated with various additional ingredients depending on the delivery format. We surveyed a large set of commercial probiotic samples (*n* = 52) to test whether products meet or exceed the labeled amount, quantitatively, and determine if they are properly labeled, qualitatively. We hypothesized that probiotic blends are formulated to have several key strains that over-represent the total CFU, while other strains are present at lower quantities. Testing the overall viable count claim on labels was completed using traditional plating and species/sub-species identity was surveyed using a novel multiplex PCR (mPCR) targeting polymorphism within the *pgi* gene. Some products also listed strain designations, which we assessed using strain-specific methods (United States Pharmacopeial Convention, 2015) for seven commonly used probiotics. We then used 16S rRNA PCR and HTS to evaluate the relative abundance of species within product formulations. Our results show that there are a select few key probiotics that account for the majority of probiotic blends and high-resolution molecular testing must replace general bacterial surveys to determine qualitative and quantitative contents of probiotic products.

## Materials and methods

### Product and standard preparation

Commercial probiotics were purchased from several retailers in Madison, WI and stored at 4°C within the end shelf-life to decrease cellular mortality. Product contents, including bacteria species, potency, capsule materials, enzymes, flavorings, and other ingredients were noted (Table S1). Samples were resuspended 1:10 (*w/v*) aseptically, remaining encapsulated when possible, and added to 1X Tris-EDTA, pH 8.0 (1X TE) (ThermoFisher p/n BP2473-1). One sample contained chocolate and was resuspended 1:100 (*w/v*) in 1X TE buffer and then incubated in a water bath at 37°C for 30 min to melt. All samples were vortexed until homogenized before further manipulation.

Samples of single-strain freeze-dried concentrates representing all 20 of the species and sub-species in the mPCR were obtained from DuPont and similarly weighed and diluted 1:10 (*w/v*) in 1X TE buffer to serve as standards for validation. The standards were previously tested with the ISO method for CFU and partial 16S rRNA sequence for species identity. Standard samples were created by combining concentrates prior to genomic DNA (gDNA) extraction (labeled with _CFU) as well as extracting each concentrate separately and then combining (labeled with _DNA), using 1X TE buffer for all dilutions. Three subsets of standards were created for each set: one with template from all samples at equal CFU (all), four standards with only the organisms in each reaction in equal amounts (rxnA-D, see Section mPCR Primer Design), and three mock communities with over-represented *Lactobacillus rhamnosus, Lactobacillus acidophilus*, and *Bifidobacterium animalis* subsp. *lactis* (Lrha, Laci, Blac). Final sample concentrations of _CFU standards before gDNA extraction were: all_CFU: 1 × 10^8^ CFU/mL of the 20 targets; rxnA-D_CFU: 1 × 10^8^ CFU/mL of the targets in each reaction; and Lrha_CFU, Laci_CFU, Blac_CFU: 1 × 10^8^ CFU/mL of key targets, 1 × 10^5^ CFU/mL of the other 19 organisms. The final concentrations of the DNA standards were: all_DNA: 100 pg/μL of the 20 targets; rxnA-D_DNA: 1 ng/μL of the five targets in each reaction; and Lrha_DNA, Laci_DNA, Blac_DNA: 1 ng/μL of key target, 1 pg/μL of the other 19 organisms. Standard concentrations are defined further in Table S6.

### Genomic DNA preparation

gDNA was extracted from 250 μL of the 1:10 dilutions of all samples and standards as described above using the MoBio Powersoil gDNA Extraction Kit (MoBio Laboratories, Carlsbad, CA) according to the manufacturer's protocol. Negative controls were included to prevent contamination of upstream testing. Individual standard gDNA was analyzed by Nanodrop spectrophotometry (ND-1000, Nanodrop, Wilmington, DE) for purity and Qubit (Qubit 2.0, Life Technologies, Carlsbad, CA) for concentration. Standards were further analyzed by electrophoresis of 2% (*w/*v) agarose gel (p/n 17852, ThermoFisher) in 1X Tris-acetate-EDTA (p/n B49, ThermoFisher) stained for 15 min with 1% (*w/v*) Ethidium bromide (p/n E-8751, Sigma) in DI water and de-stained in DI water for 15 min before visualization with UV light (Gel Logic 1500, Kodak, Rochester, NY). All gDNA was stored in −20°C until use. Statistical tests were performed using Minitab 17 (Minitab, State College, PA) and Prism 7.01 (GraphPad, La Jolla, CA). Figures were made using Prism and Geneious v. 6.1.8 (Biomatters Ltd., Auckland, New Zealand).

### Assessment of total colony forming units

Samples were resuspended 10% (*w/v*) in buffered peptone water (p/n FTPW9966, 3M) and serially diluted sufficiently to test the label claim of CFU. A pour plate technique was used, where 1 mL of the final dilution and 15 mL of deMan, Rogosa, and Sharpe (MRS) agar (p/n 288210, BD Difco, Franklin Lakes, New Jersey) supplemented with 0.05% cysteine-HCl (p/n C7880, Sigma, St. Louis, MO) were added to three replicate petri dishes. The plates were swirled gently to homogenize and cooled at room temperature until the agar solidified. Plates were incubated anaerobically at 37°C for 48 h. Resulting colonies were multiplied by the dilution factor and averaged between the replicates to give final CFU/g. This method provides enrichment for all 20 of the organisms in the following assays.

### mPCR primer design

Complete *pgi* sequences (Table S2) were extracted from both non-public DuPont culture collection genomes and those in the National Centre for Biotechnology (NCBI, Bethesda, MD) and the Genomes Online Database (JGI, Walnut Creek, CA). Sequences were categorized by species or sub-species based upon whole genome alignments and full-length 16S identity. Pairwise alignment was then performed using Geneious alignment algorithm and resulted in a consensus sequence with degenerate nucleotides representing 100% sequence identity (Figures S1A,B). All alignments were made using the default input values. The consensus sequences were then compared to the top 100 matches using the Basic Local Alignment Search Tool (*blastn*; NCBI) to locate suitable priming targets compared to the closest related sequences. Primers were designed for each species or sub-species (Table S3) and then tested for hairpins and dimers using OligoAnalyzer (Integrated DNA Technologies, Coralville, IA). Further *in silico* analysis was performed using *blastn* to prevent possible amplification of undesired targets. The assays were grouped into four pentaplex reactions (rxnA-D) based on amplicon length (Figure S2). Oligos were obtained from IDT and rehydrated with 1X TE buffer to a stock concentration of 100 μM and stored at −20°C.

### mPCR validation

Reactions were optimized for primer concentration, annealing temperature, MgCl_2_ concentration, dNTPs, and GC enhancer by gradients (data not shown). All primer combinations were tested individually against all other 19 species and sub-species to assess non-specific primer binding. Primer target specificity was further validated by testing each primer set against up to 10 different strains of each species and sub-species. Limit-of-detection (LOD) and preferential amplification experiments were tested using the standards as listed above. The mPCR reaction formula and thermocycler settings are listed on Table S4.

### Probiotic sample testing by mPCR and strain-specific PCR

Samples and standards were tested according to the PCR procedures listed in Table S4. Amplicons were visualized using 2% agarose gel electrophoresis with ethidium bromide staining as described above or by 2% E-Gel with ethidium bromide (p/n G600002, Invitrogen). Samples requiring sequence confirmation were cleaned with PCR Clean-Up and Gel Extraction Kit (Clontech, Mountain View, CA) and sent to Eurofins Genomics (Eurofins MWG Operon LLC, Louisville, KY) for Sanger sequencing. Sequences were analyzed using Geneious.

### High-throughput 16S rRNA gene sequencing

Samples and CFU standards were processed using a custom barcoding scheme as previously described (Caporaso et al., 2011). Briefly, triplicate PCR was performed with the 16S rRNA V4 primers in Table S3 and associated Golay barcodes according to the PCR procedures listed in Table S4. Amplicons were visualized using 2% agarose E-Gels with ethidium bromide, normalized with SequalPrep Normalization Kits (p/n A1051001, Applied Biosystems), pooled and concentrated with Microcon 30K Centrifugal Columns (p/n UFC503024 EMD Millipore, Merck KGaA, Darmstadt, Germany). The amplicon pool was sequenced using 2 × 250 Paired-End Illumina MiSeq technologies (Pioneer, Johnston, IA) with the addition of 25% PhiX to increase library diversity. Sequencing data was processed using the Quantitative Insights into Microbial Ecology (QIIME v1.9.1) pipeline (Caporaso et al., 2010). Reads were paired using fastq-join (Aronesty, 2011) and filtered to remove reads that contained ambiguous bases or a Phred quality score <30. The remaining sequences were clustered *de novo* at 100% identity with uclust (Edgar, 2010) and assigned a taxonomic identity using the default Greengenes database (DeSantis et al., 2006; v 13_8) in QIIME. Additionally, taxonomy was manually assigned to *de novo* OTUs representing >0.1% of the total reads by pairwise alignment to the closest type strain in the EZ-Taxon database (Kim et al., 2012) to achieve greater accuracy and resolution (Figure S4). Phylogenetic trees were generated using Geneious.

## Results

### Total viable count in samples compared to expiration date

**Figure 2:**
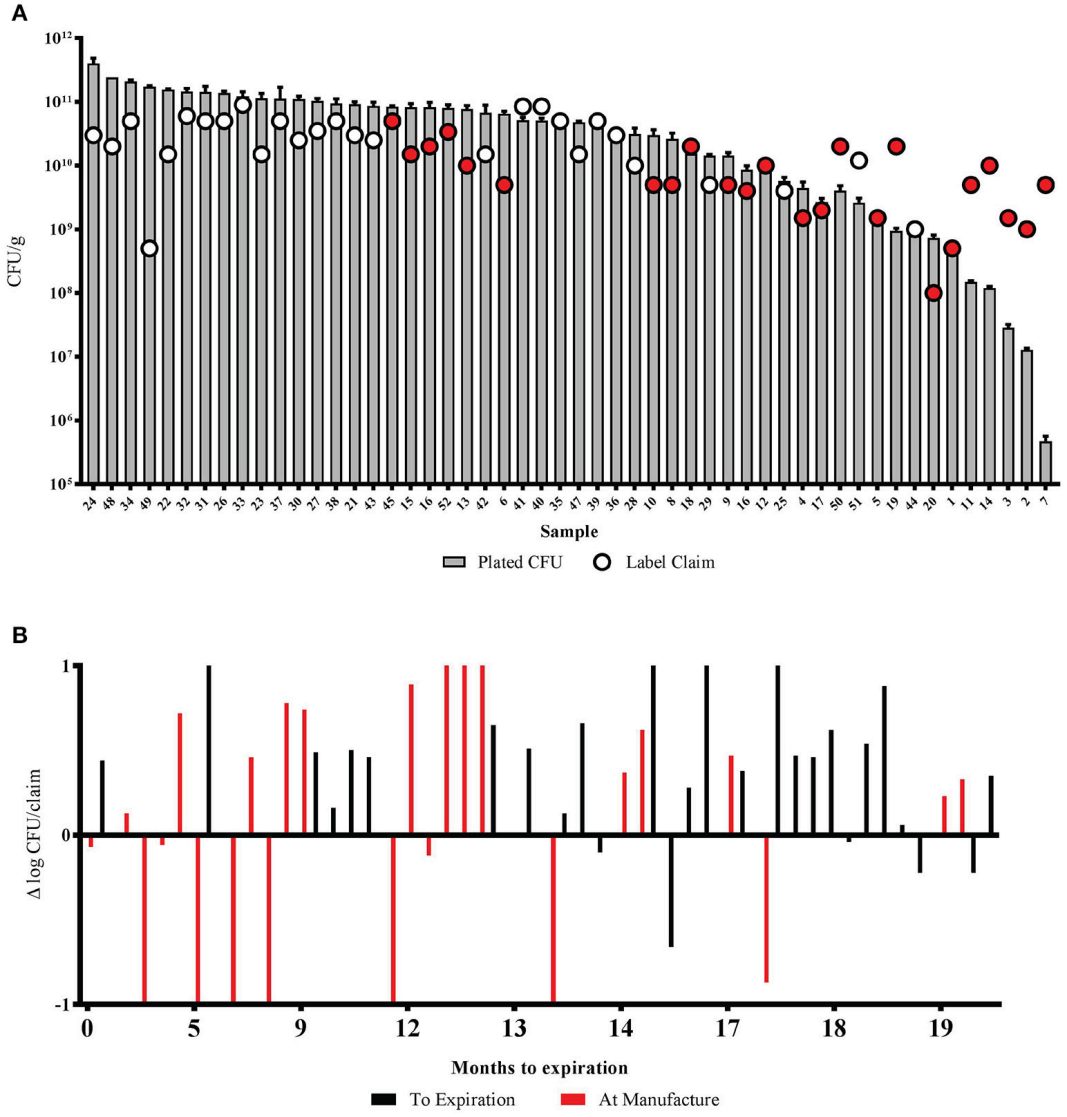
**Total colony forming units of probiotic products compared to labeled potency. (A)** The CFU of each sample compared to the label claim. Samples are organized by decreasing total CFU/g. Error bars show the standard deviation of each triplicate plate count. **(B)** The months until expiration is noted on the horizontal axis. Samples above the 0 y-axis gridline are above label claim, and those below are below label claim. All samples in red claimed potency at the time of manufacture.

The products listed an array of ingredients including capsule type, excipients, and specialty ingredients such as flavoring, vitamins and minerals, and enzymes such as lactase, lysozyme, and protease (Table S1). The average labeled count was 2.3 × 10^10^ CFU/g, with minimum and maximum counts of 1.0 × 10^8^ CFU/g and 9.0 × 10^10^ CFU/g, respectively. Many of the products (*n* = 24) listed the disclaimer that potency measurements on the label were made at the “time of manufacture,” and are represented in red in Figure 2. The plated count had an average of 6.6 × 10^10^, with a minimum count of 4.7 × 10^5^ and a maximum count of 4.0 × 10^11^. Overall, 35 of the samples (67.3%) had total CFU above the label claim, and four of those samples had an excess of over 1 log (Figure 2A). Furthermore, products quantified at time of manufacture had significantly less average CFUs than those labeled “to expiration” (*p* = 0.015, 2-Sample *t*-Test) and were trending toward having more samples below the label claim (*p* = 0.080, 2-Sample *t*-Test). The time to expiration from date of measurement was also noted, although there was no correlation between overall time to expiration and congruence with label claim (Figure 2B).

### mPCR validation

Primers were validated against multiple strains of each sub-species noted in the assay. The *B. longum* and *B. infantis* assays were compared to the BLIR test (Lewis et al., 2015) using gDNA standards and both assays successfully differentiated the sub-species (Figures S1C–E). Each primer set was tested against all other species and sub-species standards to confirm no cross-amplification. Some of the higher G+C templates in bifidobacteria did show faint non-specific binding, although amplicon sizes were distinguishable. Furthermore, *in silico* analysis showed that the *B. infantis* and *B. breve* assays may amplify other species of bifidobacteria that are not typically sold as probiotics. All results were considered positive only if the gel bands exactly matched *in silico* amplicon length.

Measurement of the individual standard gDNA extractions showed that on average, 1.2 × 10^10^ cells produced a yield of 51.8 ng/μL of high molecular weight DNA with an average A280/A260 nm of 1.93. One sample had 2.1 × 10^8^ CFU and had a gDNA yield of 11.6 ng/μL. No significant sampling yield bias was seen between the lactobacilli and bifidobacteria standards (One-way ANOVA, *p* = 0.261). The all_CFU controls amplified in all reactions, showing that there is no inhibition of individual reactions when all are blended at similar concentrations. The all_DNA control was serially diluted to 1 pg/μL and all but one reaction amplified. This adheres to the definition of LOD as the lowest concentration at which 95% of positive samples are detected (Bustin et al., 2009). The Lrha_CFU, Laci_CFU, Blac_CFU controls all amplified the labeled species, however only 78.9% of the lower dilutions amplified for the _CFU samples and none of the _DNA lower dilution targets amplified. This shows that high concentrations of single organisms can have an inhibitory effect on targets with lower concentrations. Considering the above controls, the mPCR assay is effective for blends of the listed target bacteria that are at 1 pg/μL, or 2.3 × 10^5^ cells of starting material, which is below most recommended effective probiotic doses.

### Accuracy of bacterial species labeling in probiotic products

**Figure 3:**
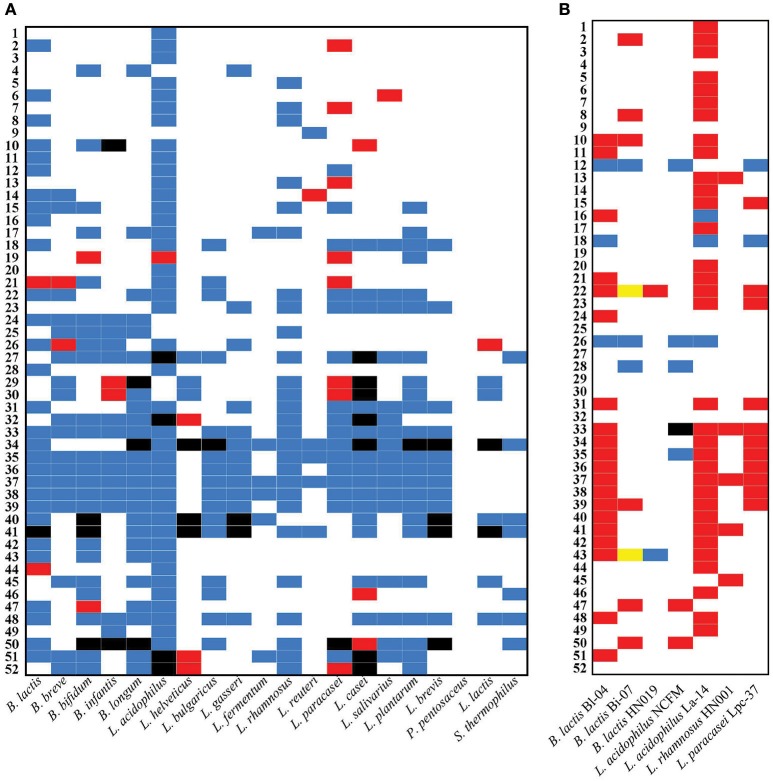
**PCR assay of species, sub-species, and strain identity compared to label claim**. The presence of organisms is visualized for **(A)** the mPCR and **(B)** the strain-specific PCR assays as different colors: blue denotes claimed and present; white denotes not claimed and absent; red denotes not claimed and present; black denotes claimed and not present; yellow denotes strain-specific assays unable to be fully characterized using the present assays. Samples are ordered based on species taxonomy by 16S rRNA sequence.

Probiotic samples were tested and assessed by comparing resulting amplicons to positive control ladders (Figure S2). Discrepancies from the label claim were retested to rule out PCR error. All amplicons were compared further against the 16S profiling and all results were mapped based on the label claims (Figure 3A). Overall, 11 (21.1%) of the blended products with at least two organisms had one or more claimed probiotic organisms missing or too diluted to detect. Conversely, 18 (34.6%) blended products had an additional organism not listed on the label claim. Considering some samples had both unlabeled positives and labeled negatives, 22 (42.3%) of the samples tested showed evidence of having incorrectly listed the target species and sub-species, only two of which did not originally claim one of the target genus *Bifidobacterium*. Some samples appeared to have switched some species, such as *B. infantis* for *B. longum* (29 and 30), *L. paracasei* for *L. casei* (29, 30, and 50), and *L. helveticus* for *L. acidophilus* (32, 51 and 52). Noteworthy, some of these closely related species have been historically difficult to distinguish until recent advances in molecular biology.

### Detecting strains in blended products

In general, 18 (34.6%) of the products listed specific strain designations. Samples that matched the species of the target strains were tested with the strain-specific primers listed in Table S3 and are visualized in Figure 3B. Two of the samples (33 and 44) were confirmed to have incorrect *L. acidophilus* strains labeled. Four samples (33, 42–44) incorrectly labeled the presence of *L. acidophilus* strain NCFM, where it was not found to be present. The majority of products (36/41) that contain an *L. acidophilus* were confirmed to have strain La-14. Two samples (22 and 43) had multiple *B. lactis* strains indistinguishable by SNP typing. Some products listed strains that do not have USP reference methods and therefore their strain designation could not be confirmed.

### Relative abundance of organisms in the product microbial blends

**Figure 4:**
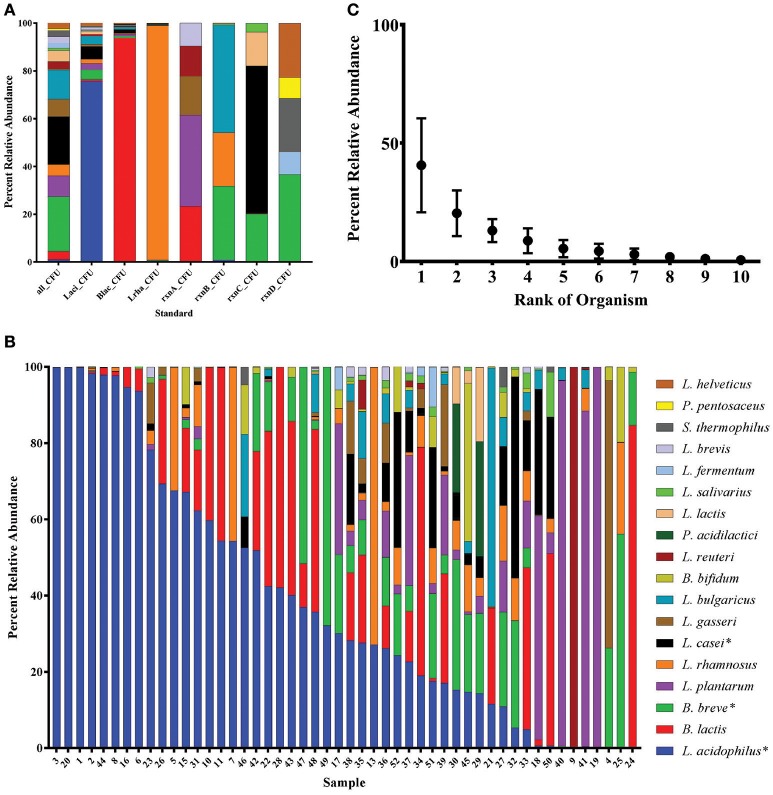
**High-throughput sequencing of the 16S rRNA gene in probiotic products**. Bar graphs show the percent abundance of **(A)** controls and **(B)** commercial samples. Filtered reads are not shown. OTUs that represent more than one organism have asterisks by the species name. Samples are ordered by decreasing key species: first *L. acidophilus*, then *B. lactis*, and *B. breve* abundances. **(C)** Thirteen of the samples had 10 or more probiotics detected using the mPCR assay. Each OTU in the samples was ranked from high to low abundance regardless of identity, and the averages are noted as dot plots with error bars representing the standard deviation.

We focused analysis on OTUs comprising more than 0.1% of total sequencing reads which resulted in 42 OTUs that were grouped to type strains using EzTaxon (Table S5, Kim et al., 2012). Most species were individually distinguishable except for several closely related species: the *B. breve* group that included *B. longum* and *B. infantis;* the *L. casei* group that included *L. paracasei;* and the *L. acidophilus* group that included *L. helveticus*. The average reads per sample after quality filtering was 58,144 reads and only one sample (14) had <10,000 reads and was removed from analysis. Controls were also sequenced to assess the accuracy of the abundance calculations (Figure 4A). Comparisons of standard CFU dilutions to percent reads resulted in a linear regression line with an R-squared value of 89.6% (simple regression). HTS results were ordered based on the two most abundant species overall in all tested products, namely *L. acidophilus* and *B. lactis*, which represented 35.6 and 15.7% of all sample reads, respectfully (Figure 4B, Figure S3). *L. acidophilus* was significantly more abundant than all other species, even after removing samples positive for *L. helveticus* using the mPCR, while *B. lactis* was significantly more abundant than all species but *B. breve, L. plantarum, L. rhamnosus, L.gasseri*, and *L. reuteri* (*p* <0.05, Tukey's multiple comparisons test). Furthermore, the two species on average made up 65.8% of the reads in blends with at least two probiotics. The abundances of the top 10 probiotics in each product positive for 10 or more probiotics with the mPCR test were assessed. The average abundance of the top OTU group in each product by input was nearly two orders of magnitude higher than the 10th ranked OTU group (40.7–0.6%, respectfully), and the general decrease in product abundance fits a decreasing logarithmic curve (*R*-squared 92.6%, simple regression; Figure 4C).

## Discussion

The strain-specific health benefits and safety of probiotics are of utmost importance to dietary supplement industry leaders, researchers, regulatory entities, and consumer groups. Current identification methods are not amenable to mixed microbial communities and therefore probiotic bacteria must be correctly identified prior to blending of multiple strains across genera and species. Although efficacy research on probiotics is increasing, without correct identification and labeling strains cannot be associated with specific studies. Additionally, this makes it difficult for consumers to choose products based on health claims associated with specific strains. With increasing focus on the small genomic variations that differentiate strains, and the potential metabolic repercussions therein, there is a great need to use high-resolution genotyping methods to assure identity as well as quantity. Recently, the International Probiotic Association approved Flow Cytometry as a method for enumerating probiotic bacteria (International Organization for Standardization, 2015). This method quickly detects viable cells to satisfy the traditional definition of probiotics as “live microorganisms which, when administered in adequate amounts, confer a health benefit on the host” (FAO-WHO, 2001), but does not differentiate cells based on genotype (Davis, 2014). Colony morphologies of closely related species and sub-species will not suffice for distinguishing the very closely related probiotic bacteria, making DNA-based methods the best option for classification.

Several other molecular methods have been evaluated for probiotic testing, including qPCR (Postollec et al., 2011) and microarrays (Patro et al., 2015). While many of these reports have identified incorrect labeling of probiotic organisms, no single test has been proposed to survey mixed microbial finished goods using a single gene target. One report from the FDA recognized this technology gap and introduced a test based on shotgun sequencing, and utilizing a custom in-house bioinformatics pipeline (Patro et al., 2016). While metagenomic sequencing can offer the resolution needed to identify all strains within a sample, simpler methods like PCR provide more resolution than current standards, with less expense and time to release. An industry accepted intermediate method must affordably, rapidly, and accurately provide species-level resolution regardless of product formulation.

In this study, we sought to understand the baseline of regulatory compliance by investigating label claims of 52 commercial probiotic products for quantity and genetic identity. Culture-based plating methods showed that a majority of products (*n* = 35) contained total amounts of viable probiotics above the label claim, which is higher than a previous report (Weese and Martin, 2011). Perhaps not surprisingly, products quantified at time of manufacture showed less overall CFU, and many were below label claim well before the listed expiration date. Our novel mPCR assay enabled relatively rapid detection of 20 distinct probiotic species and sub-species. The authors acknowledge that as new species of probiotics are introduced, they may or may not contain *pgi* genes and that these additions will need to be designed and validated to flexibly fit with the method described herein. This new method revealed identification discrepancies for 22 of the 52 products, several of which were likely due to misidentification of sub-species. While labels may be technically correct in identifying a species, it is important to denote the correct sub-species. For example, *B. infantis* is often used as a dietary supplement to establish infant microbiota in the presence of human milk oligosaccharides, a function that has not been demonstrated by *B. longum* (LoCascio et al., 2010). Some labels acknowledged incorrect classification, such as sample 49 that read “*B. infantis* (*B. lactis*)” which is scientifically incorrect and likely confusing to consumers. While few products listed the strain content on the label, the strain-specific USP testing demonstrated that it is possible to identify highly clonal strains using traditional PCR methods, and is verifiable when indicated. Finally, the HTS based on 16S rRNA gene sequence showed an uneven abundance of probiotics in blended products, where the most dominant strains, particularly *L. acidophilus* and *B. lactis*, represented over half of the reads in all of the samples.

These results clearly show probiotic strains in these dietary supplements were characteristically not of equal distribution, similar to results demonstrated previously by HTS (Patro et al., 2015) and microarray analyses (Angelakis et al., 2011). Although the CFU testing only determined viable cells, PCR can successfully amplify intact extracellular DNA or DNA from dead cells (Kramer et al., 2009), so the abundance of viable cells for the different probiotics could be different. As mentioned, many different methods are available to determine viable amounts of specific species (Davis, 2014), although it still remains difficult to quantify mixed microbial constituents in routine industrial and commercial product quality assessment. While this study does not provide an ideal solution to measure viability of different strains, it does highlight a gap in methods to determine the shelf-life stability of individual strains after they have been combined into a commercial mixture.

Although not demonstrated here, we hypothesize that manufacturers are perhaps formulating products based on the stability or cost of particular strains, or even on consumer awareness of select species. While formulations of input strains seemed skewed for high abundance of a few species, sequencing also demonstrated that the ingredient strains seem to be free of any other microbial contamination, including any pathogenic species, filtered at 0.1%. While not comprehensive, 46 of the commercial probiotic products we surveyed included more than one organism, further highlighting the need for a technique to determine each of the major input organisms at the species level.

Having established that a significant proportion of commercial probiotic products do not meet basic requirements of the correct taxonomic group (mostly at the species level) listed on the ingredients list, we developed methods that enable the industry to identify and release probiotic products. These methods will also help formulate, blend, and label probiotic products to meet the necessary standards for the regulatory agencies and consumer groups alike. As the health-promoting roles of bacteria become more substantiated, and the biochemical functions attributed to microbiomes advance toward therapeutic applications, it will be paramount to use sound, state-of-the-art, and affordable methods to formulate commercial products and document their composition.

## Data deposition

The HTS data has been deposited in the Sequence Read Archive in NCBI under accession number SRP090599 and in Qiita as ID 10681. Proprietary *pgi* gene sequences have been uploaded to GenBank in NCBI and can be accessed using the accession numbers listed in Table S2.

## Author contributions

Conceived and designed the experiments: WM, RB, and BS. Performed the experiments: WM, AH, and BZ. Contributed analysis: WM, AH, RB, and BS. Wrote the paper: WM, RB, AH, and BS.

## Funding

RB and the Klaenhammer Laboratory at North Carolina State University are supported by a research contract from DuPont Nutrition & Health.

### Conflict of interest statement

The authors declare that the research was conducted in the absence of any commercial or financial relationships that could be construed as a potential conflict of interest. WM, AH, BZ, and BS are all employees of DuPont Nutrition & Health, which produces probiotic cultures that are used as ingredients in finished dietary supplements. Samples tested in th is report were chosen without specific knowledge of probiotic supplier, and many products do not contain DuPont strains.

## Supplementary material

Figure S1 **Multiplex assay design**. Alignments of the *pgi* genes are shown for **(A)** lactobacilli and **(B)** bifidobacteria. Primers are shown as arrows above the target sequence. Black lines in the sequence blocks denote polymorphism. Specific examples of primers are shown with **(C)** *B. longum* reverse, **(D)** *B. infantis* forward, and **(E)** *B. infantis* reverse. Strains with (T) represent the type strain of each sub-species.

Figure S2 **Visualization of the positive bands from the mPCR assay**. A virtual gel created in Geneious is next to an E-Gel® with the results of testing gDNA standards. The band identities and amplicon sizes are listed to the right.

Figure S3 **Heat map of relative abundance of probiotics in samples based on high-throughput sequencing**. The relative abundance of probiotics in each sample is visualized with shaded cells, with darker shading representing higher abundance. Blue cells represent the presence of organisms that are claimed by the products, while red cells are organisms not claimed by products. OTUs that represent more than one organism have asterisks by the species name.

Figure S4 **Mapping of the OTUs to type strains in EzTaxon**. The alignment tree that maps each of the 42 OTUs to 16S rRNA gene sequences from type strains of each of the 20 target species. Tree was generated using Geneious Tree Builder with default settings.

Table S1 **Sample contents**. Gray boxes denote a probiotic claimed in the product.

Table S2 **Strains used to develop the mPCR *in silico***. Accession numbers for whole genome sequences are noted if available. GenBank accession numbers are noted for strains without associated genome sequences.

Table S3 **Primers used in the study**.

Table S4 **Settings used for all PCR assays**.

Table S5 **HTS read analysis**.

Table S6 **Standard concentrations**.
