# Supplementary figures and images for: Genotyping by PCR and High-Throughput Sequencing of Commercial Probiotic Products Reveals Composition Biases
Source: Front Microbiol. 2016 Nov 3;7:1747. doi: 10.3389/fmicb.2016.01747 (PMC5093124; doi:10.3389/fmicb.2016.01747)

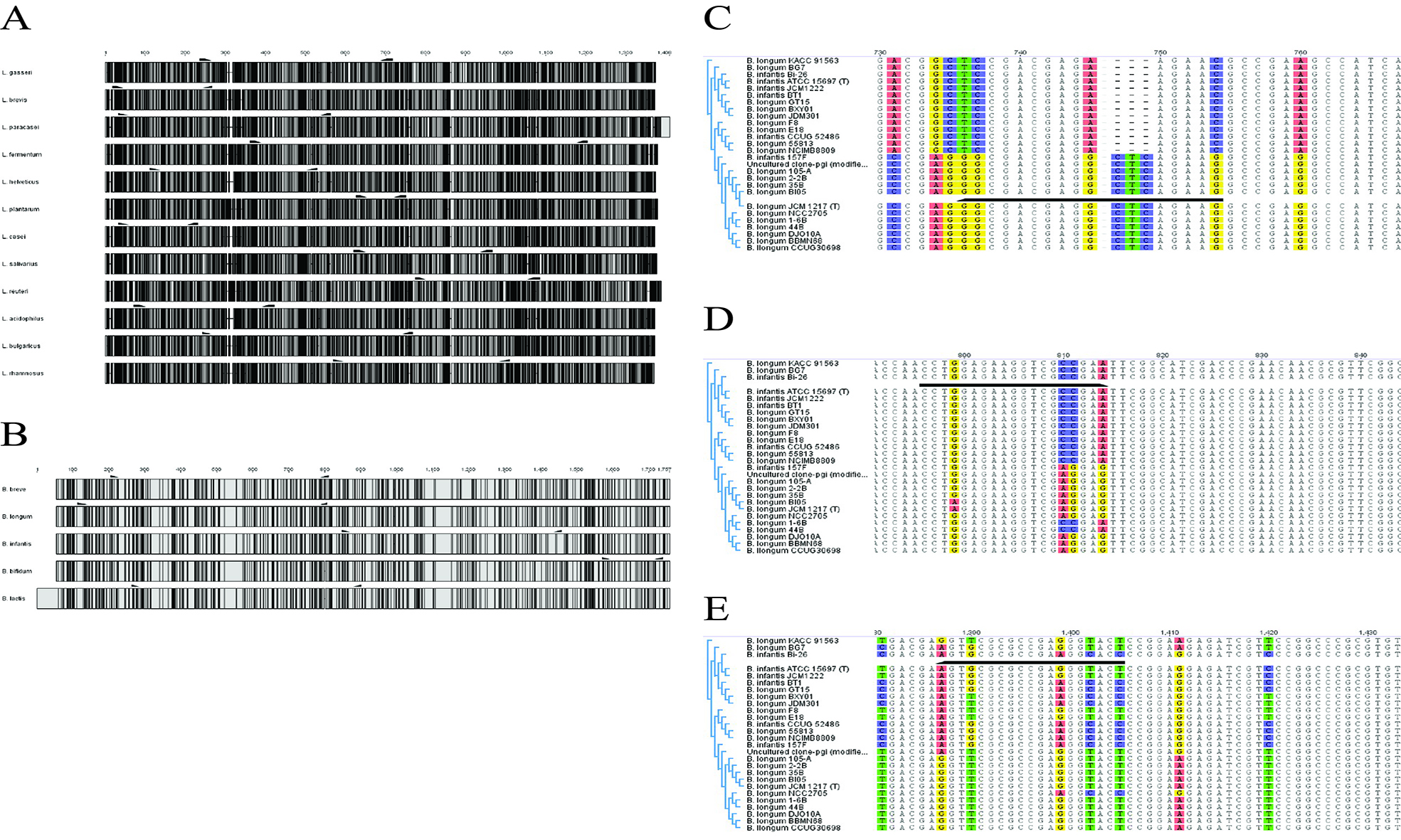

Supplement: Figure S1 — Multiplex assay design. Alignments of the pgi genes are shown for (A) lactobacilli and (B) bifidobacteria. Primers are shown as arrows above the target sequence. Black lines in the sequence blocks denote polymorphism. Specific examples of primers are shown with (C) B. longum reverse, (D) B. infantis forward, and (E) B. infantis reverse. Strains with (T) represent the type strain of each sub-species. [file Image1.JPEG]

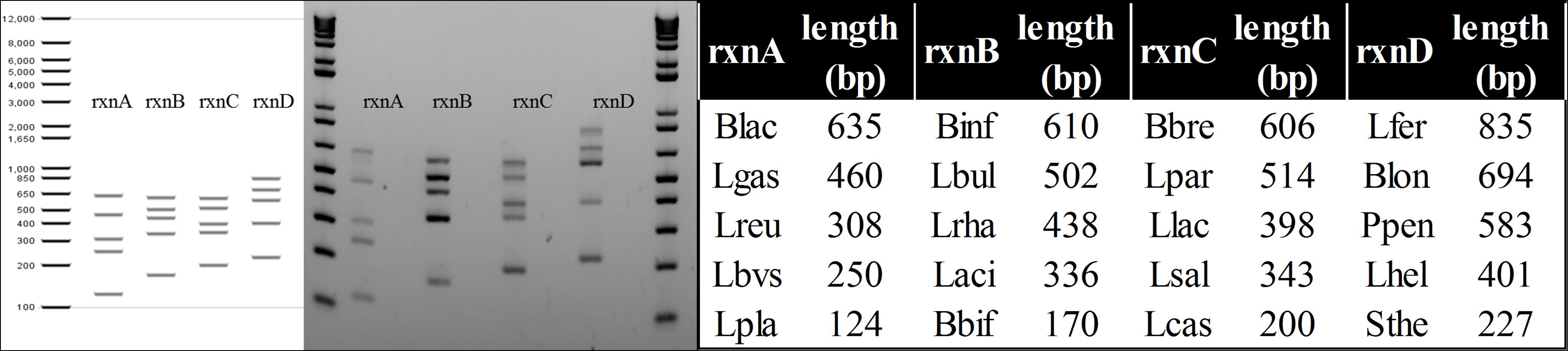

Supplement: Figure S2 — Visualization of the positive bands from the mPCR assay. A virtual gel created in Geneious is next to an E-Gel® with the results of testing gDNA standards. The band identities and amplicon sizes are listed to the right. [file Image2.JPEG]

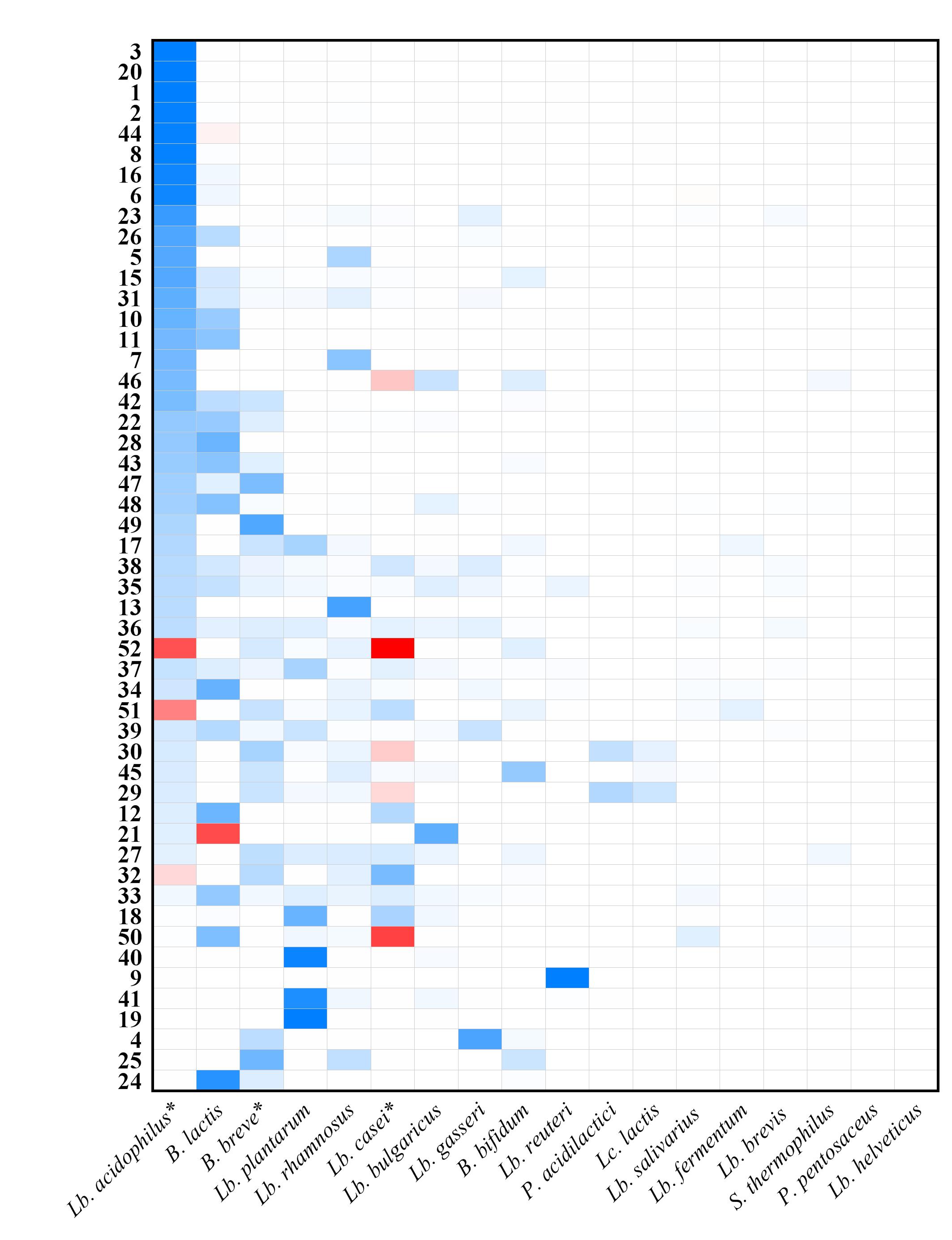

Supplement: Figure S3 — Heat map of relative abundance of probiotics in samples based on high-throughput sequencing. The relative abundance of probiotics in each sample is visualized with shaded cells, with darker shading representing higher abundance. Blue cells represent the presence of organisms that are claimed by the products, while red cells are organisms not claimed by products. OTUs that represent more than one organism have asterisks by the species name. [file Image3.JPEG]

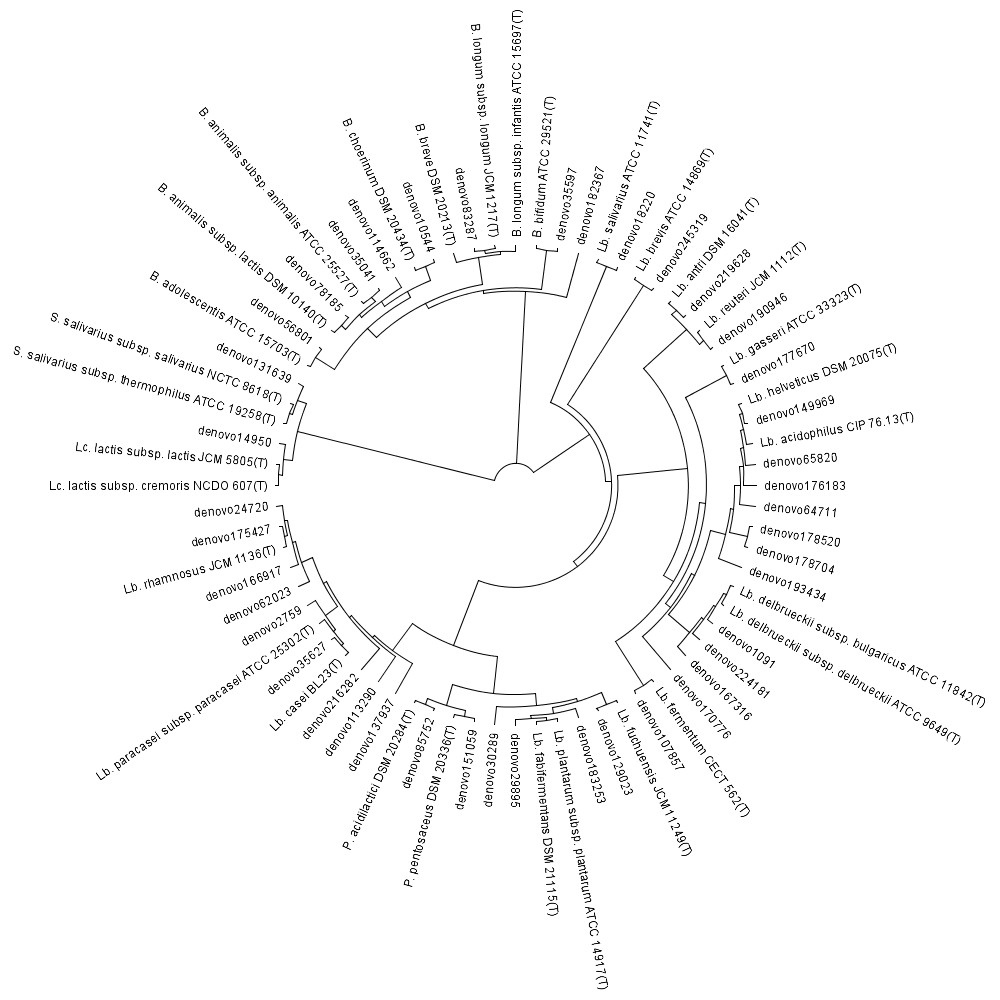

Supplement: Figure S4 — Mapping of the OTUs to type strains in EzTaxon. The alignment tree that maps each of the 42 OTUs to 16S rRNA gene sequences from type strains of each of the 20 target species. Tree was generated using Geneious Tree Builder with default settings. [file Image4.JPEG]
